# An Interdisciplinary Mixed-Methods Approach to Analyzing Urban Spaces: The Case of Urban Walkability and Bikeability

**DOI:** 10.3390/ijerph17196994

**Published:** 2020-09-24

**Authors:** Bernd Resch, Inga Puetz, Matthias Bluemke, Kalliopi Kyriakou, Jakob Miksch

**Affiliations:** 1 Department of Geoinformatics—Z_GIS, University of Salzburg, 5020 Salzburg, Austria; inga@deltami.de (I.P.); kalliopi.kyriakou@sbg.ac.at (K.K.); jakob.miksch@sbg.ac.at (J.M.); 2 Institute for Quantitative Social Science, Harvard University, Cambridge, MA 02138, USA; 3 GESIS—Leibniz Institute for the Social Sciences, 68159 Mannheim, Germany; matthias.bluemke@gesis.org

**Keywords:** wearable physiological sensors, real-time perceptions, qualitative questionnaires, geospatial analysis, urban spaces, mixed-method approaches

## Abstract

Human-centered approaches are of particular importance when analyzing urban spaces in technology-driven fields, because understanding how people perceive and react to their environments depends on several dynamic and static factors, such as traffic volume, noise, safety, urban configuration, and greenness. Analyzing and interpreting emotions against the background of environmental information can provide insights into the spatial and temporal properties of urban spaces and their influence on citizens, such as urban walkability and bikeability. In this study, we present a comprehensive mixed-methods approach to geospatial analysis that utilizes wearable sensor technology for emotion detection and combines information from sources that correct or complement each other. This includes objective data from wearable physiological sensors combined with an eDiary app, first-person perspective videos from a chest-mounted camera, and georeferenced interviews, and post-hoc surveys. Across two studies, we identified and geolocated pedestrians’ and cyclists’ moments of stress and relaxation in the city centers of Salzburg and Cologne. Despite open methodological questions, we conclude that mapping wearable sensor data, complemented with other sources of information—all of which are indispensable for evidence-based urban planning—offering tremendous potential for gaining useful insights into urban spaces and their impact on citizens.

## 1. Introduction

As functional spaces and complex systems, cities are not only places of infrastructure, communication processes, technology networks, or human agglomeration areas, but they constitute multi-dimensional conglomerates of a wide variety of (socio-geographical) processes, in which the citizens play a central role [1]. These processes reflect widespread interaction between citizens and their surrounding urban spaces in their daily life and routines.

In contrast to traditional urban research efforts, which explore the city from a systemic point of view, questions of where, when, and how people respond to and interact with their urban environments are receiving increasing attention from a subjective and quantitative methodological viewpoint. Such questions allow researchers to derive deeper insights into how well a city serves its inhabitants, particularly concerning liveability [2], quality of life [3], and urban wellbeing [4].

Consequently, citizen-centric approaches are a critical element in future urban planning and analysis because weighing all public and private interests is one of the core elements in planning processes [5]. Urban planning ideally reflects and incorporates all public and private parties, and minimizes conflicts to achieve well-balanced planning results, preferably for all citizens. Thus, all available information and knowledge sources should potentially be considered in the planning process [6]. This participatory approach is of increasing importance because citizens are becoming more emancipated and demanding and clearly articulating their claim for participation in urban planning and decision-making [7].

As part of a larger and ongoing scientific development that aims to improve the technologies used for analyzing urban space (e.g., [8]), our contribution focuses on the challenges of integrating objective data with subjective data collected from citizens. These human-centered approaches are of particular importance because understanding how people perceive and react to their surroundings depends on several dynamic and static factors, such as traffic volume, noise, personal sense of safety, the urban configuration, and greenness. Subjective perceptions are highly context-dependent and, thus, trigger situation-dependent emotional responses. Analyzing and interpreting these emotions against the background of environmental information can provide insights into the spatial and temporal properties of urban spaces [9].

Previous approaches to analyzing urban spaces have mostly focused on traditional methods such as qualitative surveys, a variety of diary-based approaches, and geospatial data analysis [10]. More recently, the use of wearable sensors has rapidly been gaining momentum in urban research [9]. For instance, integrative Global Positioning System (GPS)-based timestamped measurements with multi-sensor boards, travel diary instruments, and visualization aids (such as LifeLog and LifeLog View; [11]) offer tremendous potential for gaining new insights into the dynamics of urban spaces, such as relationships between built-environments and human behavior. Moreover, it has become possible to study brainwave patterns (EEG) to passive exposure of urban green spaces via photographic presentations in experimentally-controlled laboratory settings [12]. However, as each physiological and social-scientific method comes with its limitations, several uncertainties can compromise the analysis of urban spaces: Surveys and qualitative interviews are prone to respondents’ selective attention, and perception, faulty memory, and cognitive biases [13,14]; geospatial analysis often lacks detailed and complete geodata; and wearable sensors are characterized by a number of measurement uncertainties in data acquisition and analysis. These limitations call for an integrative approach to enhance the data purification and the analytical quality before arriving at far-reaching conclusions for urban planning (see Section 3 for more details).

### 1.1. Trends Towards Using Wearable Sensors and eDiary Apps in Urban Research

In recent years, our potential to better study and understand the environment (e.g., [11]) has dramatically increased based on the analysis of physical features using multiple sensing technologies (triple-axis accelerometer, barometric pressure, humidity, temperature, light, audio, and GPS) attached to human carriers. At the same time, human sensor data, i.e., measurements from physiological sensors to infer citizens’ emotion-psychological states like stress, relaxation, wellbeing, safety or distinct emotions, have increasingly sparked interest in urban research. Conclusions can now be drawn on urban conditions like walkability (a city’s attractiveness or its opportunity for walking) [15,16], bikeability (a city’s attractiveness or its opportunity for bicycling) [17], urban safety, and other aspects based on human experiences. Inferring emotion-psychological processes from physiological measurements is still challenging—and, surprisingly, underrepresented in the scientific literature. Methods exist that derive emotion-psychological states from measurements of physiological parameters such as skin temperature (ST), galvanic skin response (GSR), electrocardiogram (ECG), heart rate (HR), heart rate variability (HRV), Blood Volume Pulse (BVP), and electromyography (EMG). These measurement technologies are nowadays embedded in single devices with wearable wristbands or chest belts, thereby enabling minimally intrusive and cost-efficient acquisition of psycho-physiological data.

Although research efforts exist that use physiological sensors and aim to better understand urban systems, they still suffer from shortcomings concerning the analysis of wearable sensor data and unambiguously relating measured physiological states to the urban environment. At present, only fundamental insights on the analysis of physiological measurements exist, resulting from a number of basic research projects. The remaining research gaps comprise 1) the selection of appropriate physiological parameters for extracting emotional states, and 2) the combination of the chosen parameters in new sensor fusion algorithms.

Furthermore, to complement the analysis of physiological measurements, ground truthing mechanisms are necessary to relate the sensor measurements to the environment because the stimulus or context that causes a perceived emotion (i.e., the “stressor”) cannot be unambiguously identified or located in a time series of sensor measurements. This is different from using sensors in lab studies, where researchers can control the stimulus environment and their timed presentation. Therefore, participants typically have to use an eDiary app to provide more details, often on-site and in near real-time (e.g., LifeLog; [11]), followed up by post-hoc interviews.

However, retrospective (subjective) reports entail an element of uncertainty when relating the emotional states to the supposed environmental triggers. Furthermore, some erroneous cognitive processes such as introspective limits, cognitive biases or memory errors, environmental conditioning effects, and cognitive and emotional adjustment to habitually encountering stressors (among others) hamper the quality of these reports and their suitability for drawing conclusions about urban spaces [13,14].

### 1.2. A Mixed-Methods Approach to Analyzing Urban Spaces

To deal with these shortcomings, we propose an interdisciplinary mixed-methods approach that leverages the unique strengths of various methods [18]. Thereby, a single method may overcome the vulnerabilities of another method. In a more comprehensive approach, we combine various data sources, all of which produce georeferenced data: wearable physiological sensors, an eDiary app, and videos from a first-person perspective (chest-mounted camera), supplemented by personal georeferenced interviews (or standardized surveys) with typical geospatial analysis. This gives us information with higher quality compared to mere single or double method approaches. Notably, as the title of the manuscript suggests, our proposed the mixed-methods approach is suitable for analyzing urban spaces in general, while the paper demonstrates the potential of the approach in the particular use cases of walkability and bikeability. Other potential use cases include analyzing the effect of urban green spaces, urban wellbeing, barrier-free planning, and others.

Our study addresses the following research questions (RQ):

1. How can heterogeneous methods be combined or integrated for the analysis of urban spaces?
2. To what degree are the results from quantitative and qualitative approaches related to each other?
3. Are there striking similarities, or rather dissimilarities, in the results of the different methods?

## 2. Related Work

The rapid development of high-performance sensor technology has led to small and flexible wearable biosensors that allow human emotions (based on individual perceptions) to be captured by measuring physiological parameters. These sensors are useful for evaluating the walkability and bikeability of cities better than using self-reported impressions in post-hoc interviews [17,19,20]. Simple and direct sensations, together with more complex emotions, are reflected in physiological parameters that can be registered in real-time, such as ST, GSR, or HRV [21,22]. Changes in the activity of the autonomic nervous system can be measured with sensors and allow us to identify stressful events. In particular, the combination of GSR and ST provides insights into the emotional state of humans who move and feel through space.

Biosensor measurements serve as objective indicators, as they are not based on self-reports. Instead, they rely on non-reactive assessments using technical equipment (alternatively through peer or expert outsiders) collecting the data. Subjective distortions, which often occur in self-reports, are thus prevented. The main drawback of objective physiological data is that they do not permit direct conclusions to be drawn about the specific quality of human experience or behavior. This requires additional interpretation, for instance, through self-reports [23].

### 2.1. Stress Detection through Wearable Sensors

It has long been known that people respond to various stress-inducing stimuli with unspecific bodily reactions [24]. Stress is often defined as the “perception of threat, with resulting anxiety discomfort, emotional tension, and difficulty in adjustment” [25]. Cannon (1926) supposed that any deviation from *homeostasis* (the bodily “steady state”) causes the body to enter a fight-or-flight response, resulting in measurable physiological oscillations [26]. Thereby, the autonomic nervous system (ANS) reacts to stressors in a bid to re-establish homeostasis on a psycho-physiological level [27,28,29]. The associated physiological signals that reveal ANS activity—such as HRV, GSR, ST, BVP, a.o., are considered to be reliable stress indicators reflecting both the intensity and the quality of an experience, at least when calibrated against an individual’s baselines (see below) [30,31,32].

Nowadays, research efforts leverage the capabilities of wearable physiological sensors to detect signals of stress because they excel in comparison to traditional laboratory equipment regarding the amount of collected data and portability of the devices. Biosensors provide high temporal resolution resulting in continuous, high-quality measurements that are accurate, timely, detailed, and retain contextual information, especially when coupled with location tracking technologies such as the Global Positioning System (GPS) [32,33,34]. In contrast, one-time measurements such as interviews, even fine-grained self-reporting via questionnaires or apps, cannot provide the high temporal resolution of wearables [35].

However, some preconditions must be met to obtain reliable and useful measurements for stress detection. First, the sampling frequency needs to be sufficient to accurately depict the signal. Second, proper placement of the wearables is necessary to avoid ambiguities and to appropriately record the physiological signal. Despite these prerequisites, the signals vary based on the natural oscillations of the physiological status of the human body. All of these fluctuations are inevitably recorded. To obtain more accurate data, filters are needed that remove the noise [36,37]. The nature of the signal and the type of noise are some of the criteria for selecting optimal filters. To remove the ambient noise (e.g., shifting signal levels, alternatively peaks/spikes), filters such as the Kalman filter, Butterworth filter, Wavelet Decomposition, among others, have been devised [38]. Wearable biosensors coupled with GPS provide valuable information about a subject’s urban context and the emotions associated with it at a high spatiotemporal resolution. This synergy offers great potential for better understanding cities as complex systems [35].

### 2.2. The Potential of Wearables for Urban Analysis

Over the last decade, more and more approaches have integrated wearables and emotional experiences in urban analysis coupling “spatial-emotional data”. This provides tremendous potential for better understanding urban areas. Nold (2009) first introduced the idea of emotional cartography, combining objective physiological measurements with location data as a new kind of psychogeography [39]. The author built a customized portable and wearable “Bio Mapping” device that recorded GSR and GPS data. In addition, participants could vocalize their mental state and describe what happened during their walks. The outcome was a map that depicted all the collected data, creating an “emotion surface” that blends objective emotional states with subjective experiences [39].

Nowadays, numerous research efforts leverage the capabilities of wearables for urban analysis, but they vary in terms of (1) the methods applied, (2) the physiological signals recorded, (3) the type of physiological signals analyzed, and (4) the sample size (see Table 1).

Similarly, Layeb and Hussein (2016) investigated the urban soundscape and its characteristics that most frequently led to stress [40]. Bergner et al. (2013) combined wearables with cameras to investigate the stress states of pedestrians [41]. Layeb and Hussein (2016) recorded only GSR and used the “StressPhaseIdentifier software” to detect stress phases [40]. Chen et al. (2016) also used wearables and cameras, but primarily investigated the variations of physiological signals, namely, ECG, EMG, GSR, and ST [42]. Zeile et al. (2016) revealed the most common triggers for cyclists using wearables, cameras, and a smartphone-based application that allowed users to create entries about their perceived emotions occurring in the field, based on variations in their personal GSR and ST measurements [20]. Their study was the only one that combined these methods. Additionally, another study combined physiological sensors and first-person perspective videos in a virtual reality environment [43].

In conclusion, most previous studies used a limited set of methods (mostly analysis of measurements from wearables and/or questionnaires at the end of a field test), and all investigated the variations in GSR for detecting stress [44,45,46]. The one exception is [17], who recorded both GSR and ST and detected stress through a specific algorithm they had developed. Birenboim (2019) also used wearables and questionnaires but scrutinized the variations of GSR and HR to detect stress [35].

Our study goes beyond the state of the art in that it combines several techniques in a mixed-methods approach. We aim to deliver more reliable and comprehensive results because the used methods can complement each other and partially mitigate their respective shortcomings.

## 3. Methodology: A Mixed-Methods Approach for Analyzing Urban Spaces

Evidently, researchers felt an increasing need to combine methods before arriving at firm conclusions based on wearables alone. It appears that better algorithms for extracting stressor-related information only addresses part of the problem of correctly attributing physiological signals to the environment. These physiological measurements can be complemented and validated by continuous subjective impressions reported via an eDiary app, as well as first-person perspective cameras and retrospective interviewing.

**Figure 1:**
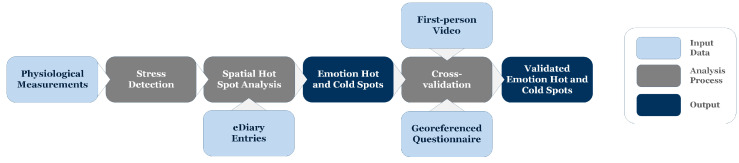
Workflow of the presented mixed-methods approach.

We propose a comprehensive mixed-methods approach (see Figure 1), combining methods from various disciplines (geoinformatics, computer science, social science, and psychology) to leverage the strengths and overcome the vulnerabilities of each method. We combine typical geospatial analytics with the analysis of physiological signals measured by wearable sensors, eDiary app entries capturing immediate impressions reported by study participants, and videos from the participant’s first-person perspective (a chest-mounted camera), complemented by georeferenced interviews (or standardized surveys) to achieve higher data quality compared to previous approaches. Notably, all used datasets are explicitly georeferenced and contain a timestamp, which constitutes the common ground for their combined analysis.

The mixed-methods workflow shown in Figure 1 integrates several georeferenced data sources. The first step in the workflow is to collect physiological measurements, from which we extract moments of stress that study participants experience (see Section 3.1). After that, we perform a spatial hot spot analysis using a specific algorithm according to [47], integrating the physiological measurements with eDiary app entries in a cartographic representation. This analysis results in spatially fine-grained emotion hot and cold spots that identify places of clustered stress and clustered relaxation. In the next step, we perform a cross-validation of these hot and cold spots, (1) by visually identifying the cause of moments of stress from video footage (first-person camera), and (2) by qualitatively comparing responses to a georeferenced questionnaire. Thereby, videos from a first-person perspective function as a cross-validation of the presumed emotional triggers because they facilitate memory recall and situational recognition, can strengthen traces of emotion memory, and help identify or prevent misattribution of feelings to the wrong environmental stimuli. In fact, the videos serve a dual purpose: (1) cross-validating eDiary entries that may be subjectively biased, and (2) adding context (“ground-truthing” the stressor) and quality (type) of emotion to sensor data. The latter are high-resolution time series data, but provide no context information and are thus “semantically poor”. The developed methodology is interdisciplinary (embracing urban research, geoinformatics, and social-scientific methodologies) and technology-supported (geospatial analyses, human sensor technologies, videos), clearly going beyond the current state of the art in the analysis of urban spaces.

In addition to qualitative surveys and geospatial urban analysis, the core element of innovation herein is the unique combination of human sensor technologies and wearables (to detect changes in physiological parameters as a response of the human body to an emotion-psychological process, which is located in a specific physical environment) with eDiary apps and first-person videos for the analysis of urban spaces and cross-validation of the different approaches. The geolocation of these measurements allows us to verify where people responded to an emotional stimulus, and this geoinformation can then be linked to the corresponding urban environment, which needs to be augmented by a subject’s perception of the environment.

A vital benefit of combining these methods is that ambivalent situations can be identified and explained: Either different participant groups respond differently to the environment (stressed vs calm), or participants themselves are ambivalent about a situation, but their focus rests on a particular stimulus or environmental element. Different methods allow conflicting responses to be identified, and high-resolution sensors allow quick emotional shifts to be detected, which may escape participants’ attention.

To position the mixed-methods approach presented herein within the mixed-method literature, several similarities but also differences need to be discussed. “Mixed-methods” approaches combine quantitative and qualitative data analysis [48,49]. More specifically, a methodological “triangulation” [50] is needed to maximize the validity of data, or filter out the relevant data from the noise surrounding the data that is not relevant for answering the research question. Unlike sequential designs, our approach does not specify either a full quantitative → qualitative or qualitative → quantitative sequence of methods. The openness of the individual experiences of subjects moving through space, and a deeper understanding of unspecific physiological parameters, call for a parallel mix of methods in the analysis of emotional responses to the environment, be it a static environment or a continually changing environment for people who are mobile. If anything, of all designs discussed in [51], our design mostly resembles the Sequential Explanatory Design regarding data collection, in that it seeks additional confirmation over and beyond the continuous sampling of quantitative data obtained from the biosensors. However, conceptually the design is closer to the Concurrent Triangulation Design, because separately analyzed data have to be merged or integrated, and then interpreted jointly.

Arguably, the idea that validity can be maximized after combining methods that originate in different domains is a contentious one. In the present case, we indeed look not only at sources of information *complementing* each other but also at (the possibility of) *eliminating errors* and *preventing erroneous conclusions* by blending them. Neither of the methods is dominant from the outset. Two examples may demonstrate this. Assume a dangerous situation emerged for a cyclist due to the poor conditions of the cycling lane. Physiological signals may reflect a continuous activation of the nervous system, but the respondent—paying more attention to the traffic than to the ground—has hardly noticed his or her struggles. Self-report would be introspectively limited. Likewise, even if a stressful situation were reported, it may be the case that a subject attributes arousal to the size of a crossing, while the video camera may show that a problem exists only if ongoing traffic is blocking the cycle lane. Precisely because time stamps and geolocations allow the same phenomena to be described from different angles, the multi-method approach is suitable for increasing validity. All three fundamental principles of mixed-method triangulation apply; namely, converging information, diverging information, and information complementing each other.

From a methodological point of view, no objective reference or methodological gold standard (“truth”) exists, against which the results of any method could be compared. Each method yields a particular set of information, each of which requiring situation-specific interpretation, that is, a “qualitative” integration of partially quantitative data. Furthermore, exact replicability of results is in most cases an unattainable scientific goal. Therefore, generalizing statements about (objectively predictable, scientifically expected, and consistent) relative advantages of any method or combined methods are impossible. The data (physiological measurements, eDiary entries, video sequences, responses, to the questionnaire, etc.) as well as the investigated real-world settings are highly time- and context-dependent, and in fact unique. This fact prevents that any comparison between two situations (or samples or methods) can yield the same (objective and replicable) advantage of one method (or a combination) over another. Hence, there is an urgent need for methodological triangulation and ground-truthing in a mixed-methods approach.

### 3.1. Extracting Emotion Information from Wearable Sensor Data

We assess the physiological signals resulting from perceptions and emotions of pedestrians using wearable biosensors. Based on technology evaluation and laboratory tests [52], we recommend the Empatica E4 (a wristband measuring GSR and ST) and the Zephyr Bioharness (a chest strap measuring a variety of cardiological parameters like ECG, HRV, and others). Our sensor fusion method extracts emotion information (moments of stress) from the measured data using a customized signal analysis procedure [53].

First, a low-pass filter (cut-off frequency 0.5 Hz) eliminates high-frequency variations in measurements that may be caused by technical inaccuracies, followed by a high-pass filter (cut-off frequency 0.05 Hz) to filter the tonic GSR as an indicator of each participant’s baseline. Then, our rule-based algorithm detects patterns in the measurement data that are presumed to indicate a physical stress reaction: 5 s of GSR increase, followed by a temporally delayed (+3 s) decrease in ST. Next, the algorithm looks for a local maximum, followed by a local minimum in ST together with a slope steepness in the GSR increase of ≥10° [53]. The algorithm reliably identifies moments of stress in the measured physiological parameters at an accuracy of 84%, as shown in a validation study (see [53] for more details). We acknowledge, though, that no scientific consensus regarding which of the physiological parameters should be integrated has yet been established. Furthermore, some detailed questions about the specific algorithmic setup remain unresolved. 

### 3.2. eDiary App Entries and First-Person Perspective Videos

To complement the sensor measurements with subjects’ perceptions of the environment, we use an eDiary app developed by geographic information scientists (GIScientists) together with psychologists and urban planners. It allows the users to record which events occurred and what they encountered while moving through urban space [54]. Augmenting sensor data with subjective elements captured through the eDiary app is essential because the signal analysis procedure can merely identify moments of stress, but not their emotional quality (which type of feeling) or the urban context (what real-world trigger instigated an emotion). Thus, the eDiary entries help interpret the measurement time series by infusing human-generated meaning and observations, which are elicited through (perceptions of) the environment.

The eDiary app used in this study [54] offers a simple multi-screen interface, where each screen can contain one or more questions (e.g., open, closed, checkboxes, radio buttons, intensity sliders). The questions and interface are easily configurable through a configuration file, according to the particular study’s needs. For the studies presented in this paper, we designed a three-screen layout asking for the following inputs: (1) the quality of the perceived emotion (happiness, anger/disgust, sadness, fear); (2) the context or cause of the triggered emotion (cars, bicycles, green space, people, sights, cityscape, weather, intersections); and (3) the intensity of the felt emotion.

In principle, eDiary applications are a step forward in that they represent multiple-wave designs that allow for repeated and more detailed questioning around the time and near the location of potentially stressful events. They can be designed with experimenter-triggered prompts after fixed periods of time (interval contingent design); alternatively, respondents may enter data regarding their experiences whenever appropriate, i.e., whenever they experience a stressor (signal-contingent design). The latter design is more flexible and probably more suitable for urban analysis. However, this model also carries the risk of introspective limits, that is, participants overlooking or downplaying relevant cues that should have triggered subjective records [55]. For instance, there is positive–negative asymmetry, which may lead to underrepresentation of positive environments in the subjective record [56,57]. Because of such limits, physiological reactions may correct memory distortions and help identify dangerous situations in space that a participant hardly noticed consciously. Alternatively, physiological reactions can also identify positive, relaxing situations that simply escaped participants’ attention before a record could be made.

In addition to the physiological sensors and the eDiary app, participants are equipped with a wearable video camera (GoPro), attached with a chest strap, to record the real-world situation from the perspective of a test person. The video recordings serve as a visual verification aid for the sensor-derived stress moments. Furthermore, they allow for more certain conclusions on the actual stressor that may have caused the stress reaction. Currently, we manually extract sequences from relevant real-world situations captured by the video footage in the lab after the field-phase, because analyzing videos in a dynamic urban setting would be highly challenging and is out of scope for our research.

### 3.3. Combined Analysis in a Visual Analytics Approach

We synthesize the moments of stress extracted from the objective sensor data from wearables with the subjective data from the eDiary app to visually check for spatial co-occurrences (ground-truthing). This allows us to identify—and spatially locate—triggers of the detected moments of stress. This step contributes to the analysis of urban space by revealing spatial accumulations of human physiological reactions of pedestrians (or cyclists) to the urban environment.

Currently, we pursue a visual analytics approach to assess the commonalities and differences between the geospatially analyzed sensor measurements and the eDiary entries, which are both aggregated to raster cells and standardized over the number of measurements and eDiary entries in a given area. Even though the eDiary entries cannot be considered a reliable gold standard as such—due to a variety of errors and biases inducing uncertainty such as blurred memory, dilutions due to cognitive processes, such as self-censoring rather than proving spontaneous reports, environmental conditioning effects, and habituation, the illusory truth effect, and others [58]—they show a different spatial emotion distribution compared to the sensor measurements. Thus, the two data sources complement each other [10]. Potential further developments for the current visual analytics approach are described in Section 5.1.

### 3.4. Georeferenced Questionnaires

Questionnaires pose a series of questions to gather information from respondents [59,60]. They are the most useful and versatile method of eliciting subjective data about people’s experiences. More specifically, autobiographical memory is relevant for recollecting specific episodes or personal events, which are typically charged with valence [61]. Despite the advantages, the study of autobiographical memory has been criticized for the lack of accuracy in retrospective reports (e.g., [62]), and it has to be granted that it is difficult to control for error and bias in self-reports. Close timing after an event and additional memory aids (such as maps and further cues) can help reduce the impact of false memory reports on findings [13,14].

With proper geocoding, it becomes possible to locate the respondents’ answers in space. If multiple geocodes are available, multiple subjective reports may be gathered and, thereby, reveal information about the environmental context about which questions may be asked (alternatively about the contextual influence on any kind of subjective answers). However, even when geocoded, the utility of retrospective (post-hoc) interviewing is limited, as it does not cover all the experiences that matter in urban analysis. First of all, practical reasons limit the number of questions that can be asked. Furthermore, the high temporal resolution required also far exceeds typical autobiographical memory capacities (for exceptions, see [63]).

For our walkability study (see Section 4), we designed a 19-item questionnaire, ranging from personal background information (urban/rural living situation, age, education level, household type, employment status), mobility behavior (frequency of physical activity, physical mobility restrictions, car ownership, public transport use), attitude towards walking (motivation for walking, e.g., health, joy, necessity), and subjective walkability assessment (availability of sufficient space for walking, crossing streets, car drivers’ behavior, the joy of walking in a specific area, assessment of the particular urban environment for walking, general feelings). Additionally, study participants were asked to point out on a map where they felt relaxed or stressed during their test-walk and to describe the situation that purportedly caused this feeling. Our bikeability study used the same questionnaire with a specific focus on the bicycle infrastructure.

## 4. Case Studies and Results

To validate our integrative mixed-methods approach, we carried out two field studies, (1) investigating the *urban walkability* of two regional capital cities, Salzburg and Cologne (56 participants, 3–7 September, 2018), and (2) evaluating the *safety of the urban bicycle infrastructure* in Salzburg (18 participants, 30 October to 30 November, 2018). Both studies showed substantial convergence between the results of each methodological component while revealing a strong potential of the methods to complement each other. For both field studies, hot spots (spatially clustered moments of stress) and cold spots (spatially clustered moments of relaxation) identified in the human sensor data correspond to participants’ perceptions provided through the eDiary app and a geolocated (post-hoc paper–pencil) questionnaire. While hot spots (red) are to be interpreted as stress accumulation points, cold spots (blue) are considered as spots with noticeable low-stress intensity. The limitations and issues described in Section 4.1 and Section 4.2 apply to both field studies.

**Figure 2:**
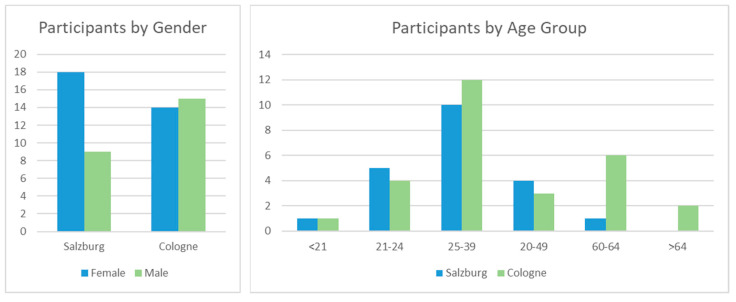
Gender and Age Distribution of the Study Participants.

For both studies, test persons were approached via email (existing email lists for empirical studies). The selected test persons were instructed about the study protocol, functionality of the wearables sensors, use of the eDiary app, and purpose of the video recording on-site before the sensors were mounted. All participants signed an opt-in agreement (informed consent) for the acquisition and analysis of their data for the purpose of this study. Figure 2 shows the gender and age distribution of the study participants. The weather during the two field studies was similar with temperatures between 12 °C and 20 °C, mostly overcast skies, and short periods of slight rain.

### 4.1. Urban Walkability

**Figure 3:**
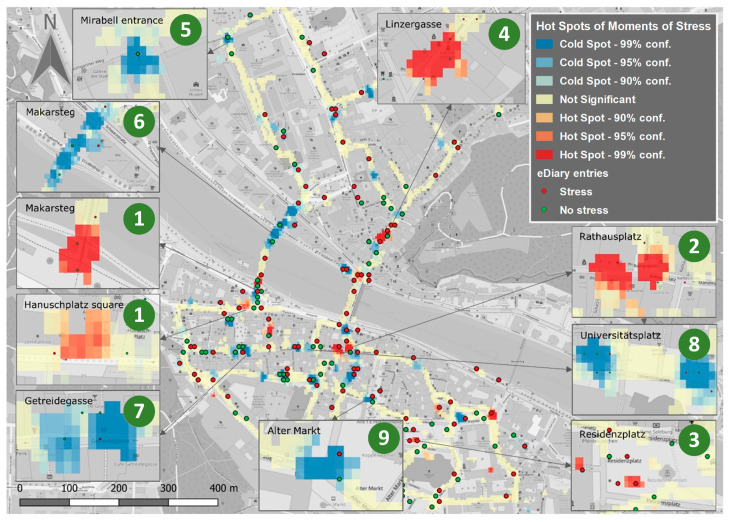
Walkability-related hot spots (stress clusters) and cold spots (areas of relaxation) in Salzburg (based on [10]).

In a first study, we investigated *urban walkability* using the approach presented above and comparing the city centers of Salzburg and Cologne. We recruited 56 participants (27 for Salzburg and 29 for Cologne) who were instructed to walk through their respective cities with sensors mounted on their bodies (Empatica E4 wristband, Zephyr Bioharness, plus GoPro first-person video camera). Subjects were also asked to enter observations, impressions and emotions into the eDiary app on a smartphone and to answer a customized questionnaire after their walk. Figure 3 shows the geospatially analyzed physiological sensor data for the city of Salzburg, whereby red areas indicate hot spots and blue areas indicate cold spots. The data was analyzed by applying Getis–Ord Gi*, a well-known geospatial hot spot analysis method.

The results identified stress hot spots near Hanuschplatz square (1), in Getreidegasse at Rathausplatz square and in front of Mozart’s birth place (2)—one of the city’s major tourist hubs—at Residenzplatz square (3), and Linzer Gasse (4). The cold spots for Salzburg were located in close proximity to tourist attractions, and moments of relative calm and lingering were particularly present when participants had the opportunity to pay attention to these sights (e.g., in traffic-calmed areas). In particular, the cold spots in Salzburg comprise locations that offer a particular exposure to the sights or offer a special view: these include the iron gate at the entrance of the Mirabell Gardens (5), at the transition to the Makartsteg (6), at Getreidegasse (7), at Universitätsplatz square (8), and close to the Residenzplatz square and Alter Markt square (9). In general, hot spots were found where pedestrian areas are characterized by special traffic dynamics such as the constant presence of pedestrian traffic (e.g., “crowds”) or the intersection of traffic lanes (bicycles, cars), forcing the pedestrian to interrupt their walk or even to sidestep.

Both the hot and cold spots identified in the human sensor data corresponded to participants’ impressions, provided through the eDiary app, and to a geolocated (post-hoc paper-pencil) questionnaire. This correspondence between methods is similar for both cities.

**Figure 4:**
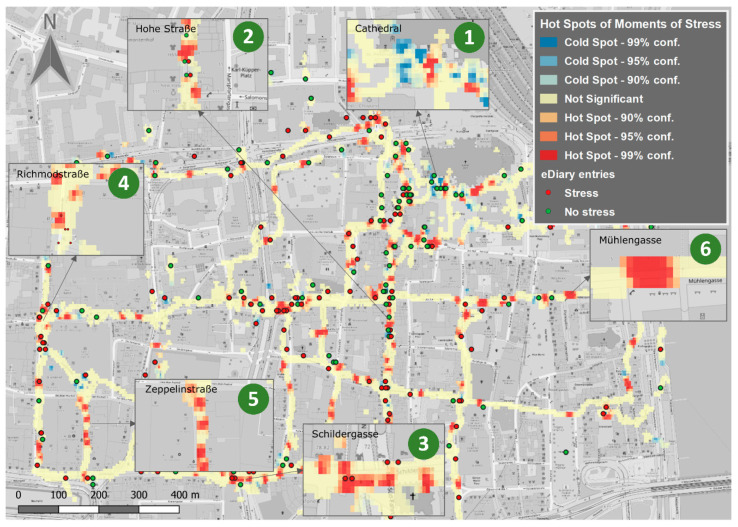
Walkability-related hot spots (stress clusters) and cold spots (areas of relaxation) in Cologne.

For Cologne, the number of hot spots is higher overall (see Figure 4): considerably more hot spots are present in the study area compared to Salzburg. These numerous hot spots can, therefore, no longer be regarded separately but need to be grouped to achieve clarity in the interpretation. The high number of hotspots for Cologne can be explained, on the one hand, by the total number of visitors covering a larger area compared to Salzburg, and, on the other hand, by the comparatively low number of tourist attractions far away from the cathedral (1), at the same time, the cathedral, as a central tourist attraction, results in a higher number of cold spots in its proximity. According to the eDiary entries, the cold spots are associated with the cheerfulness of the nearby surroundings. However, participants also repeatedly commented on “crowds” related to the cathedral. The cathedral is thus an ambivalent place in the sense of stress and relaxation. The situation is similar with the Hohe Straße (2): shop windows and shops, street artists and musicians invite people to linger, while the particularly busy dynamics on the sidewalks and in the pedestrian zones resemble a continuous stream of pedestrians. Pedestrians are forced to adapt to the dynamics of the pedestrian flow and, if necessary, to run behind the people who are heading in the same direction. This dynamic is not only time consuming and requires patience, but it may also demand collision avoidance. Similar patterns emerged for all shopping streets such as the Schildergasse (3), but also Richmodstraße (4), Zeppelinstraße (5), and Mühlengasse (6) (corner Kleine Budengasse). Individual comments point to disturbances during the walk by cyclists and motorists, and to increased risk of accident when walking with walking aids.

### 4.2. Urban Bikeability

In a second field study, we analyzed the *safety of the urban bicycle infrastructure* in a defined section of the city center of Salzburg. Concretely, the evaluation followed a two-step procedure in a pre-post analysis approach: (1) an existing bicycle lane was evaluated using the mixed-methods approach presented in this paper, and (2) after the city government had modified the width of the bicycle lane, the same evaluation procedure was carried out again.

**Figure 5:**
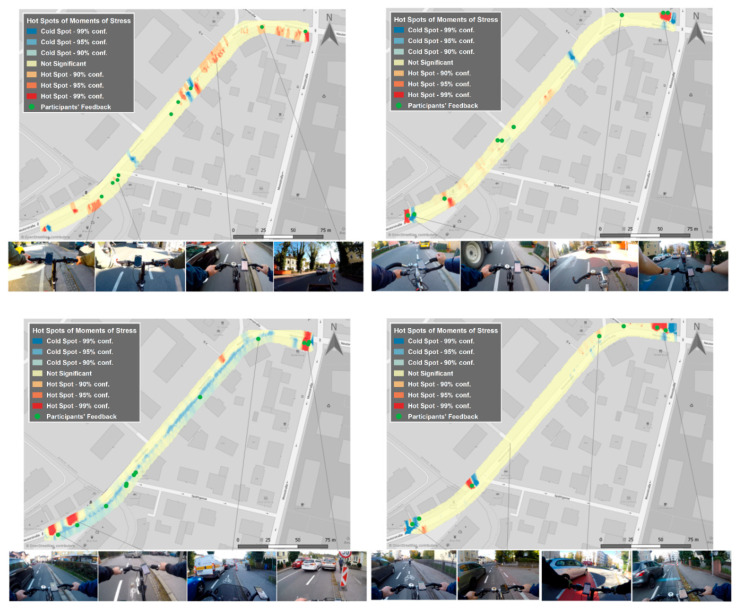
Hot spots (stress clusters) and cold spots (areas of relaxation) in the Urban Bicycle Network.

As shown in Figure 5, the stressful moments detected by wearable sensors corresponded very well to the dangerous spots identified in the qualitative survey results. However, sensor data also identified further stress points that were underreported in the survey. Additionally, most of the stress points revealed by the sensor data could be retrospectively validated with the help of first-person videos and attributed to a specific traffic situation as a cause. The localization of stress points with GPS implies that they may differ from the actual driving line due to geospatial uncertainties in the GPS positions. In terms of the effectiveness of the bicycle lane, our results revealed that in the second test phase, i.e., with a wide bicycle lane, 20% fewer stress points were detected in the physiological measurements compared to the narrow bicycle lane (12.6% less in the city, 25.9% less out of town).

## 5. Discussion and Sociological Considerations

Concerning the research questions laid out in the introductory section, we can draw the following conclusions:

RQ1: we were able to combine different and highly heterogeneous approaches and data sources by visually cross-validating the results of each method. We were thereby able to “ground-truth” the detected (wearable sensors) and reported (eDiary app) moments of stress and relaxation with real-world situations captured by the first-person cameras. An automated, quantified methodology to combine the data sets or information layers in an integrated analysis algorithm is a matter of future work.

RQ2: both hot spots and cold spots identified in the human sensor data mainly corresponded to participants’ impressions provided through the eDiary app and a geolocated (post-hoc paper–pencil) questionnaire. An approach for quantitative comparison is yet to be developed because the entropy of the different outputs is difficult to quantify, and the semantic information level of the outputs varies. Therefore, information fusion algorithms need to be investigated to merge the different information layers.

RQ3: it is striking that the different information layers exhibit similar spatial patterns (see, for instance, Figure 3). However, in some cases, these layers contradict each other. For instance, the moments of stress (MOS) derived from (objective) physiological sensor measurements are only partly in line with the (subjective) eDiary entries. These contradictions may be caused by multiple factors, including cognitive biases and errors concerning the eDiary app entries and answers to the questionnaire-items, or uncertainties in the MOS detection algorithm. Ultimately, unambiguously attributing the discrepancies is difficult because no gold standard exists against which the outputs of each method can be compared and validated. To the extent that participants produce similar discrepancies, this may instill trust in the reliability of findings.

### 5.1. Methodological Issues

Mixed-methods approaches combine quantitative and qualitative data analysis [48,49]. More specifically, a methodological “triangulation” [50] is needed to maximize the validity of data, or filter out the relevant data from the surrounding noise that is not relevant for answering the research question. Our mixed-methods approach relates to the extant literature but does not fit exactly in any of the mixed-methods categories described in [51]. More work is needed that address the methodological considerations of mixed-methods approaches in GIScience.

First of all, stress levels are only one indicator in the entire set of indicators for the assessment of urban spaces. This is particularly true for the two case studies presented in this paper (walkability and bikeability), which have traditionally been examined by analyzing multi-indicator systems. Our vision for using stress levels and emotions in the investigation of urban spaces is twofold. (1) Either stress levels can complement existing indicator-based approaches, thus acting as another indicator that can be analyzed in conjunction with other factors. (2) Alternatively, they may in the future even be able to replace indicator-based analysis, assuming that the indicators sum up the predictive power of all other indicators. To date, neither of the two options can be ruled out, and further research is needed to investigate the relationships between stress/emotion levels and other indicators.

From a methodological survey point of view, it is apparent that past studies in urban research paid limited attention to questions of sample size and composition. Not only do small sample sizes prevail, but often the composition appears to be rather ad-hoc. Urban researchers would profit from considering questions such as the following in more detail: should locals, tourists, or both, be included in a study? Is the distinction relevant, or is it rather the knowledge about the space under scrutiny, that is, being familiar or not familiar with the locations? Should the sample sizes be planned such that each subgroup is large enough to be reliably measured to provide its own analysis and allow comparisons between several groups? Is age a relevant variable for stratification of the sample, or should a stressor-related analysis collapse over participants of different age? Is there a particular motivation behind including only one gender group? How can one alleviate the concern that one tech-savvy group may be more inclined than others to use trackable devices and participate in urban studies?

Regarding the reliability of the analysis, further questions may be asked about the design of the parcours. Is there a difference between using a predetermined (fixed) path-design in comparison to a volatile (self-directed) path in a city? Can the criteria for choosing among the two designs be spelled out a priori? What about the reliability of the analysis of the two approaches? Logically, accumulating evidence is more reliable the more pedestrians or cyclists pass the same (hot or cold) spots. Thus, this approach would yield a more accurate picture of the urban environment and its affect-eliciting properties. How can one clearly distinguish between the low reliability of participants’ subjective reports on the one hand and transient emotional responses from a few affected individuals on the other hand? Is there a way to differentiate between temporally occurring phenomena that affect the majority of the sample (for instance, an unexpected traffic jam after an accident) and stable features of the built environment (narrowing of two lanes)?

From a data analysis viewpoint, drawing reliable conclusions from physiological data outside of controlled environments (labs) is difficult, despite the maturity of technological developments. On the one hand, real-world experimentation compromises reproducibility (compared to lab studies), and results in higher complexity due to additional external factors; on the other hand, the test persons’ awareness of being participants in a study may induce a variety of cognitive biases [58]. Since the analysis of sensor data does not produce unambiguous results, its combination with an eDiary app, which acts as a subjective feedback method, is necessary to determine the context and trigger of the pedestrian’s perception or emotion. The eDiary app validates the measurements for each participant, constituting a ground-truthing mechanism for the physiological data measured by wearable sensors. However, the eDiary can effectively not be considered a true gold standard because of its spatial and temporal sparsity compared to the wearable sensor data. Further research in combining hot spot maps derived from physiological sensor measurements with eDiary app entries comprise (1) the automated correlation between the two datasets in, for instance, difference maps, spatial cross-correlation or covariance-based measures, and (2) the semantic fusion of information, i.e., finding overlapping and complementing information in stress/relaxation hot spots identified in the sensor data and eDiary app entries through geostatistical methods.

### 5.2. Data and Privacy Issues

Due to the rapid development of sensor technology, there is an increasing trend in science in using spatiotemporal data from wearable sensors. However, the use of such sensor data may pose a significant risk to privacy, mostly because practitioners and the public may not be fully aware of the potential disclosure risks associated with these datasets [64]. Regarding the use of participatory sensor data in research studies, Resch (2013) emphasizes the obligation of practitioners to deal with various data protection aspects such as data ownership, accessibility, integrity, liability, and activation or deactivation options [65]. Although a large number of problem-oriented publications on the disclosure of geoinformation are available, no clearly defined solutions have been proposed [10]. Kounadi and Resch (2018) outline a geo-privacy guideline for participatory sensor data that covers the entire process chain of a research campaign [66].

To select and highlight one issue, it may seem reasonable to conclude that having multiple participants walk or cycle an identical researcher-determined path reduces the risk of de-anonymization compared to monitoring subjects following their usual path to the workplace each morning. However, in combination with time stamps and other information requested or created by the electronic device used by participants (e.g., mobile phone with apps other than the eDiary app for the study purpose), four chunks of a route may be sufficient to determine an individual’s identity [67]. When combined with other personal information, such as attitudes or economic background questions, this poses a risk to data privacy. It should become the norm that self-reported sensitive data (e.g., mobility preferences, general attitudes, health-relevant information) and inconspicuous geolocations must be stored independently at separate locations. Only researchers with (restricted) access to the table that provides links between independent IDs from both datasets should be able to run a full geospatial analysis, especially if the data are meant to be shared (e.g., as public use files [68]). While this procedure is (becoming) the norm for social surveys, it cannot yet be assumed to be fully observed in GIScience, although increasing efforts are being made in the community [69]. However, as the sample sizes grow, and as the value obtained from having access to more and more unique information about participants increases, this will become a prerequisite in the future.

## 6. Conclusions

Our presented approach advances the analysis of urban places towards a more comprehensive understanding of cities and relies on the combination of various georeferenced data sources. These data sources comprise wearable sensors and eDiary apps, together with first-person video, traditional geospatial analysis methods and georeferenced questionnaires. Advances in biosensor technology enable us to record and map the changing physiological reactions of citizens in specific real-world situations. Based on these human reactions, personal impressions and elicited emotions can be identified in designated areas. Merging “objective” measurements and other methods (qualitative questionnaires, feedback via eDiary apps, geospatial analysis, and video footage) allows conclusions to be drawn about the triggers of perceptions and emotions as reactions to the specific urban environment.

The mixed-methods approach presented in this paper can be seen as an evidence-based methodology for extracting stress hot and cold spots from physiological measurements, and combining them with other methods of urban planning. From a planning perspective, this will potentially constitute a useful “mood sensor” for future planning processes to complement traditional surveys with a dynamic layer in the planning processes [7].

The number and location of reliably identified hot spots in a city may foster a better understanding of the city as an environment for citizens and tourists. For instance, urban planners may want to relate hot spots to existing reports about traffic accidents and then take countermeasures that address the concerns reported by pedestrians and cyclists. Another example might be to create larger zones of calmness or liveliness or to follow up on newly shaped zones of interrupted or uninterrupted fluent movement to evaluate and improve the walkability and bikeability profile of the city. Furthermore, the approach is well suited for performing a pre-post analysis of measures realized in the urban mobility infrastructure, i.e., to evaluate and assess the impact after the implementation of a change in the infrastructure. It is evident that further scientific investigations and evaluations of urban planning measures are required to evaluate the impact of urban spaces on citizens.

In this regard, the combination of physiological measurements with other, more traditional datasets can be considered a valuable source of information for urban planning. This is particularly the case as urban planning is oftentimes still a closed communication process between local governmental stakeholders and not an open, transparent procedure that discusses, merges and follows the requirements of citizens and civic interest groups [7]. Ideally, all arguments should be collected and weighed up in urban planning procedures. However, in current deductive planning practice, which city governments typically initiate and implement, citizens’ requirements are often not sufficiently heard and considered. This includes particular interests, unclear planning targets, and unrealistic demands [70].

In contrast, public participation may encourage democratic processes, increase acceptance through better transparency, contribute to the establishment of more accurate and comprehensive repositories of citizens’ needs, produce an evidence-based legitimation of particular planning measures, and reduce the costs of planning process [71]. This is of utmost importance in increasing discussions about how policy-makers can foster participation and integrate the public into decision-making processes. A central question is how people can be proactively engaged to participate in these processes and how these alternatives to traditional means of participation can be leveraged in practice [7].

In this regard, our mixed-methods approach can deliver new insights into peoples’ feelings and expectations concerning their city. In contrast to previous top-down processes, our research pursues a bottom-up and inductive approach. The advantages are manifold: first, bottom-up processes are citizen-driven, where the data acquisition of urban phenomena is supported by researchers. Second, as “prosumers” of planning-relevant data, the citizens not only act as data producers but also provide central inputs for new planning issues [7]. Thus, our approach may contribute to fostering such self-organizing processes of assessing urban spaces.

Finally, our approach is not to be understood as a generic tool for solving all planning issues, but it may be able to foster another, unseen view and a more accurate understanding of the urban environment. It would be beneficial if these new insights could be integrated as indicative information in official planning processes [5]. Ultimately, we strive to provide evidence-based data to underpin the increased plausibility and relevance of our results by considering the spatial environment and its effect on citizens. Although our real-world case studies yielded plausible results, further research challenges were identified. Determining the parameters for the detection of emotions is a relevant challenge for research. Data and privacy-related issues must be taken into consideration and should be addressed comprehensively. Finally, this kind of research will contribute additional benefits to walkability/bikeability research by unraveling new perspectives derived from biosensor data never seen before.

## Figures and Tables

**Table 1:** Summary of literature review on strategies for combining other methods with wearables for investigating emotional experiences in urban areas.

Authors	Methods				Wearable Sensors		Sample
	Vocalize Description	Camera	eDiary	Survey	Physiological Signals	Type of Analysis	Number of Participants
Nold (2009)	x				GSR	Physiological signal variation	-
Bergner et al. (2013)		x			GSR	Stress PhaseIdentifier software	7
Chen et al. (2016)		x			ECG, EMG, GSR, ST	Physiological signal variation	4
Layeb and Hussein (2016)	x				GSR	Physiological signal variation	13
Zeile et al. (2016)		x	x		GSR, ST	Physiological signal variation	12
Fathullah et al. (2018)					GSR	Physiological signal variation	9
Osborne et al. (2018)				x	GSR	Physiological signal variation	30
Shoval et al. (2018)				x	GSR	Physiological signal variation	68
Birenboim et al. (2019)				x	GSR, HR	Physiological signal variation	15 (only males)
Werner et al. (2019)				x	GSR, ST	Custom algorithm	21
Zeile and Resch (2018)		x	x		GSR, ST	Physiological signal variation	2

Abbreviations: GSR: Galvanic skin response; ST: Skin temperature; ECG: Electrocardiogram; EMG: Electromyography; HR: Heart rate.
